# Treatable mortality and health care related factors across European countries

**DOI:** 10.3389/fpubh.2024.1301825

**Published:** 2024-02-16

**Authors:** Aida Isabel Tavares

**Affiliations:** ^1^CEISUC - Centre for Health Studies and Research, University of Coimbra, Coimbra, Portugal; ^2^ISEG, UL - Lisbon School of Economics and Management, University of Lisbon, Lisbon, Portugal

**Keywords:** treatable mortality, health expenditures, health resources, Europe, quantile regression

## Abstract

**Introduction:**

Despite the improvements in European health systems, a large number of premature deaths are attributable to treatable mortality. Men make up the majority of these deaths, with a significant gap existing between women and men’s treatable mortality rate in the EU.

**Aim:**

This study aims to identify the healthcare-related factors, including health expenditures, human and physical resources, and hospital services use associated with treatable mortality in women and men across European countries during the period 2011–2019.

**Methods:**

We use Eurostat data for 28 EU countries in the period 2011–2019. We estimate a panel data linear regression with country fixed effects and quantile linear regression for men and women.

**Results:**

The results found (i) differences in drivers for male and female treatable mortality, but common drivers hold the same direction for both sexes; (ii) favorable drivers are GDP *per capita*, health expenditures, number of physicians *per capita*, and (only for men) the average length of a hospital stay, (iii) unfavorable drivers are nurses and beds *per capita*, although nurses are not significant for explaining female mortality.

**Conclusion:**

Policy recommendations may arise that involve an improvement in hospital bed management and the design of more specific policies aimed at healthcare professionals.

## 1 Introduction

In 2019, before the COVID-19 pandemic, a total of 1,015,225 people died prematurely before the age of 75 in the European Union (EU). Of these deaths about 36.5% (371,570) could have been avoided through medical interventions. These death rates are higher among men, accounting for about 56% of the cases compared with 44% for women. This gap between women and men in treatable mortality is likely to influence the gap in life expectancy. The main causes of these treatable deaths include cancer, ischaemic heart diseases, and cerebrovascular diseases (1, 2). And so variations in treatable mortality, arising for instances from the treatment of cancer, may affect the gap in life expectancy between women and men [in 2019, life expectancy for women and men in EU was 84.0 and 78.5 years old (3)].

Treatable mortality refers to deaths, before the age of 75, that could have been prevented through timely and effective healthcare interventions, after the onset of diseases, with the goal of reducing case fatality (differently from preventable mortality which focus on deaths happening due to the lack of prevention, before the onset of diseases) (4, 5). Treatable mortality may be considered an indicator of the effectiveness of healthcare systems, and it is also an indicator of economic development. On the one hand, low-quality health systems are associated with high mortality (2, 5, 6). On the other hand, premature mortality implies a depletion of human capital and so a reduction in Gross Domestic Product (GDP) growth (6–9).

This indicator has taken on greater significance and become a greater priority for policy makers with the establishment of Sustainable Development Goal (SDG) target 3.4, which clearly aims to reduce premature mortality from non-communicable diseases to promote mental health and well-being (10). Therefore, improving the overall health and well-being of a country’s population must involve reducing the treatable mortality rate by ensuring timely access to care and effective healthcare interventions.

To achieve these objectives, a well-functioning health system is essential, and it relies on four basic building blocks (10–13): governance, resources generation, financing, and service delivery. While governance may be challenging to express through aggregate indicators, the remaining blocks may be reflected in some indicators. Resources generation may be described by health professionals *per capita*, financing may be captured by health expenditures, and service delivery may be expressed in indicators related to the quality of care provided in hospitals. The purpose of our work is not to monitor the performance of health systems, which is presented elsewhere (13).

The goal of this investigation is to examine health system-related factors, including finance, human and physical resources, associated with treatable mortality for both women and men across European countries for the period 2011–2019. Treatable mortality results from the absence or delayed health care interventions, and the effectiveness of these interventions, in turn, depends on the several factors that represent the building blocks of health systems. Therefore, the purpose of this work is to analyze the association of these factors with treatable mortality rates. To accomplish this, a conditional mean and a quantile panel data linear regression with country fixed effects for samples of men and women across EU countries is estimated.

**Figure 1:**
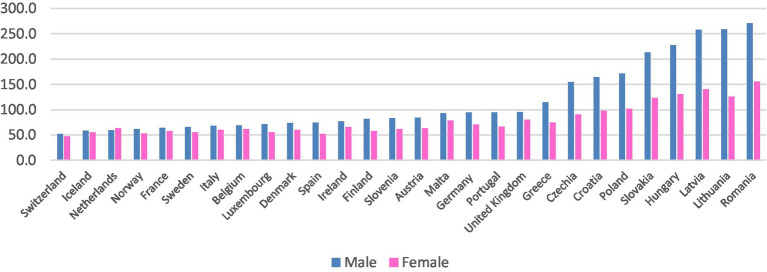
Treatable mortality rate in 2019. Annual standardized rate per 100,000 inhabitants. Eurostat (2023, online code *hlth_cd_apr*) (13); United Kingdom data for 2018.

This work addresses a gap in the existing literature on this topic and contributes to our understanding of the underlying trends that account for variations in treatable mortality across European countries (Figure 1). The findings of this study offer valuable insights for policy makers, shedding light on both the differences and commonalities between women and men in Europe. This aggregate analysis allows for international comparisons relatively to best results or practices, policies and interventions among countries, and it enables the identification of disparities and differences across countries. Among the common factors associated with treatable mortality across European countries, we have identified GDP *per capita* and health expenditures, which are important determinants for health outcomes (15–24).

Previous literature examining the explanatory factors of treatable mortality is very limited. A recent scope review performed for the period 2013–2019 did not include a single article on the determinants of health in high income countries (25). A recent and unique study (24) explored the relationship between treatable mortality, specifically related to circulatory system, and endocrine and metabolic diseases, health expenditures and GDP *per capita*. The results revealed a negative correlation between these two variables and level of treatable mortality across 37 OECD countries. A review of aggregate data studies on avoidable mortality and health services was presented in 1990 (26). Despite this review encompasses factors related to a broader concept of avoidable mortality, it identifies two general groups of factors: one related to socioeconomic characteristics and the other concerning the supply and use of health care services.

Several studies have researched the determinants of health outcomes from an aggregate perspective and have used panel data. Examples of such studies are presented in Table A1, in Supplementary Material (18–25). All of these studies explore a set of various explanatory factors, including socioeconomic factors, health care supply, and lifestyles factors to explain some outcome health variable across countries. All these studies use the GDP *per capita* as a control variable, as it has consistently been associated with health (7–9, 18–25).

## 2 Study design

### 2.1 Data and variables

Data used in this analysis is collected from the Eurostat database (14) and so it is conditioned of its availability. It accounts for 28 European countries (a list of the included countries can be found in Table A2 of the Supplementary Material and the correspondent mortality rate for 2019) for the period 2011–2019. The period begins in 2011 because it is the first year for which data is available and ends in 2019 before the turbulence of the COVID-19 years. The set of countries include both EU members, and Iceland, Norway and Switzerland which have an identical level of development to those EU members and nearly no missing data.

### 2.2 Dependent variable

The dependent variable used for this analysis is the annual standardized rate of treatable mortality of residents by sex, per 100,000 inhabitants, obtained from Eurostat table coded ‘HLTH_CD_APR’. This health outcome indicator accounts for all causes of death that can be mainly avoided through timely and effective health care intervention, before the age of 75, including secondary prevention and treatment (after the onset of diseases) (3, 4).

### 2.3 Independent variables

The explanatory variables are shown and described in Table 1 and they were selected conditional on data availability and on previous literature (11, 12, 18–26). These variables reflect the domains of financing (health expenditures), resources generation (health professionals and beds), and service delivery (inpatient related indicators) motivated by the building blocks of a health system proposed by WHO (11, 12). We use the control variable GDP *per capita* in PPP which has the label “GDPpc” (16–26). The source of all the independent variables is Eurostat database and the online code is displayed in Table 1.

**Table 1:** Description of independent variables.

Variable	Abbreviation	Description	Eurostat online code
Health expenditures in PPP as a percentage of GDP	Health_Exp	Health expenditures by government schemes and compulsory contributory health care financing schemes	HLTH_SHA11_HF
Number of physicians per 100,000 people	Physicians	Physicians practising or licensed to practice	HLTH_RS_PRS1 HLTH_RS_PHYS
Number of nurses per 100,000 people	Nurses	Practising or professionally active nurses	HLTH_RS_PRSNS
Number of beds per 100,000 people	Beds	Available beds in hospitals	HLTH_RS_BDS1
Hospital days of inpatients *per capita*	Hosp_Days_Inpat	Hospital days of inpatients refer to the total number of days that patients spend in a hospital while receiving inpatient care for all causes of diseases (A00-Z99) excluding V00-Y98 and Z38	HLTH_CO_HOSDAY and DEMO_PJAN
Average length of stay in days of inpatient (for women and men)	ALOS_m or ALOS_f	Measures the average duration of hospitalization for all causes of diseases (A00-Z99) excluding V00-Y98 and Z38. This variable takes different values for Males and Females	HLTH_CO_INPST
Logarithm of GDP *per capita*	GDPpc	Natural logarithm of Gross Domestic Product (GDP) *per capita*	SDG_10_10

### 2.4 Quantitative analysis

We start by performing a descriptive analysis of the variables. We also show the pairwise correlation between all the variables. Then, we undertake some preliminary tests: (i) a Silk-Wilkson test (27) for normal distribution of variables; (ii) a VIF estimation to check for multicollinearity; (iii) Cook’s distance to check for outliers and influential data (28); (iv) a Hausman test (29) to decide between fixed or random effects.

Finally, we obtain quantile plots for treatable mortality, and we estimate a quantile regression with fixed effects using the method of Machado and Santos Silva (30). Additionally, we used a panel data regression with fixed effects for each sample to obtain the conditional mean. A post-estimation Wald test (31) is then performed to check for global significance of the estimated models. The analysis is performed in STATA 16.

## 3 Results

We start by describing the results found in the initial tests which are needed for the following regression estimation. The pairwise correlation is presented in Supplementary Appendix Table A3, in the Supplementary Material. The Silk-Wilkson test shows that data for each variable does not follow a normal distribution (Supplementary Appendix Table A4) justifying the use of a quantile regression approach; the estimation of the VIF values confirm the absence of multicollinearity because the values are under 10 (Supplementary Appendix Table A5); the computed Cook’s distance indicates the absence of influential data; finally, despite the inconclusive results for the Males sample, for the Females sample the Hausman test showed that fixed effects model is better than a random effects one (Supplementary Appendix Table A6). As one would expect, there are fixed effects emerging from the country’s characteristics and we assume that an identical result would emerge in the Males sample, should the sample be larger. The description of the panel data statistics is presented in Supplementary Appendix Table A7. These preliminary tests and analyses lend support to the upcoming estimation.

In the Supplementary material (Graphs A1, A2), we present the quantile plots for Male and Female Treatable Mortality. It is clear from the Male graph that there is a break before the 75th quantile. Such a pattern is much less clear for the Female sample. To allow for comparison between Males and Females, we chose the 20th, 50th and 70th quantiles to estimate the quantile regression. Supplementary Appendix Table A8 displays the countries and corresponding quantiles for treatable mortality rate in 2019. Quantiles represent the varying distribution of the treatable mortality rate across countries, with lower quantiles indicating lower mortality rates and higher quantiles corresponding to higher mortality rates.

We now proceed with the presentation of the results obtained from both the panel data linear regression estimation for the mean with country fixed effects, and the quantile regression description (Table 2).

**Table 2:** Regression results.

	**Males**							
	Mean		Q 20th		Q 50th		Q 70th	
	Coef.	*P* > *t*	Coef.	*P* > *t*	Coef.	*P* > *t*	Coef.	*P* > *t*
GDPpc	−48.262	0.000	−54.916	0.000	−49.066	0.000	−43.700	0.002
Health_Exp	−3.325	0.039	−4.902	0.044	−3.516	0.046	−2.244	0.339
Physicians	−0.095	0.057	−0.070	0.192	−0.092	0.017	−0.112	0.030
Nurses	0.016	0.053	0.019	0.035	0.016	0.010	0.014	0.098
Beds	0.164	0.000	0.162	0.000	0.164	0.000	0.165	0.000
Hosp_Days_Inpat	0.000	0.294	0.000	0.315	0.000	0.119	0.000	0.205
ALOSm	−2.602	0.059	−2.876	0.042	−2.635	0.009	−2.414	0.076
_cons	588.705	0.000						
Nr Obs	180		180		180		180	
Wald Test	*F*(27,145) = 208.91	0.000	chi2 (7) = 121.80	0.000	chi2 (7) = 245.45		chi2 (7) = 141.22	0.000

**Females**								
	Mean		Q 20th		Q 50th		Q 70th	
	Coef.	*P* > *t*	Coef.	*P* > *t*	Coef.	*P* > *t*	Coef.	*P* > *t*
GDPpc	−34.355	0.000	−30.954	0.000	−34.071	0.000	−37.139	0.000
Health_Exp	−2.999	0.001	−2.234	0.057	−2.935	0.001	−3.625	0.003
Physicians	−0.088	0.002	−0.084	0.003	−0.087	0.000	−0.091	0.002
Nurses	0.002	0.686	−0.001	0.857	0.002	0.715	0.004	0.486
Beds	0.040	0.007	0.038	0.053	0.040	0.007	0.042	0.043
Hosp_Days_Inpat	0.000	0.253	0.000	0.197	0.000	0.055	0.000	0.125
ALOSf	−0.779	0.310	−0.727	0.289	−0.774	0.133	−0.821	0.250
cons	469.320	0.000						
Nr Obs	180		180		180		180	
Wald test	*F* (27, 145) = 131.85	0.000	chi2 (7) = 218.98	0.000	chi2 (7) = 410.08	0.000	chi2 (7) = 240.58	0.000

Value of *p* of ‘0.000’ means value of *p* ‘<0.0001’.

As expected, countries with larger GDP *per capita* tend to register lower treatable mortality rates, both for women and men. Equally, countries allocating large funding to health, register lower mortality rates.

The findings for the different quantiles of male mortality show that for the 20th quantile, the number of physicians and hospital days are not statistically significant. While the average length of stay in hospital decreases mortality, the number of beds and nurses, surprisingly, represent a positive correlation. This positive correlation is maintained across the other quantiles, except for the 70th quantile, where the variable number of nurses loses statistical significance. The median quantile (50th quantile) shows the highest number of statistically significant variables, the exception is the average length of stay in hospital which has no explanatory power. The highest quantile (70th quantile) has the smallest number of significant variables, specifically, the GDP *per capita*, number of physicians, and number of beds *per capita*.

Concerning the findings for women across the different quantiles, it may be said that in general there is not big difference across the explanatory power of the different variables. Economic variables (GDP *per capita* and health expenditures) and health care variables (number of physicians and beds *per capita*) are significant in the three estimated quantiles. The remaining variables, specifically number of nurses *per capita*, hospital days of inpatients, and average length of stay in hospital are not statistically significant.

Finally, comparing the results between men and women we may conclude that on average there are more significant drivers of treatable mortality for men than for women, despite the direction found for the estimated coefficients being the same for both genders. The main difference between male and female results is that for females, nurses and average length of stay is not significant. Comparing the quantiles estimations, for men the median quantile shows an identical performance with the mean and the extreme quantiles showing some differences in the significant variables. Female quantiles estimations are more consistent over the whole distribution.

## 4 Discussion

### 4.1 Key findings

The key findings of this research may be listed and described as follows: (i) there are differences in factors explaining male and female treatable mortality. However, there are also common ones that show the same direction for both sexes. (ii) The favorable drivers, which lower mortality, are GDP *per capita*, health expenditures as share of the GDP, number of physicians *per capita*, and the average length of stay in hospital for men. (iii) The unfavorable drivers, meaning those that contribute to growing numbers of treatable mortality, include nurses and beds *per capita*, however nurses are not significant for explaining mortality among women. (iv) Lastly there is a greater diversity of factors of the male distribution of treatable mortality compared with that of the female sample. However, there is some consistency in the significant related factors of treatable mortality across this distribution.

### 4.2 Interpretation

We begin by analysing the mitigating factors of treatable mortality.

Firstly, GDP *per capita* is a well-recognized driver of diminishing mortality (7–9, 32, 33) and also of treatable mortality (21, 24, 33). The mechanisms sustaining such relationships are well-described in empirical studies and may be regarded as capital accumulation (4, 7–9, 34–36): (i) improved living conditions, (ii) better access to health care, (iii) improved health care infrastructure, and (iv) higher wages and better education.

Secondly, the increase in health expenditure as a share of GDP also mitigates treatable mortality (19–24, 32, 36–38). This is related to the abovementioned effects. With an increase in resources directed at healthcare, there is an improvement toward universal health coverage, better preventive care, and chronic disease management.

Thirdly, our results point to a correlation between a higher number of physicians *per capita* and a lower rate of preventable mortality. However, this result does not show agreement across different studies, with results being mixed. There are studies that do not find any relationship (39–41), while others find a negative effect (42), while still others find that the number of physicians have a beneficial effect on health by reducing amenable mortality (43–48). The reasons for our results may lie in features coupled with increased time spent attending to patients, enabling the early detection of diseases, dedicating, and providing better treatment, and managing better acute and chronic diseases.

Fourthly, the average length of stay in hospital improves the treatable mortality for men, but not for women. There is not a consensus surrounding this result as it is linked to several factors (49, 50). There may be a need for closer surveillance and patient control, and it is beneficial for men to stay longer in hospital from an aggregate point of view.

Next, we explain the factors that contribute to a worse treatable mortality indicator across countries.

Firstly, the number of nurses *per capita* is associated with the male mortality rate, but not the female one. Although this result is contrary to the findings of some studies (51, 52), our results are more aligned with others (53–55) which point to the number of nurses being below a threshold level and the increase in the patient–nurse ratio. Despite the disparities of nurses across Europe, there is a general tendency for staff shortages (56, 57) which may not be locally observable in some countries or hospitals, but it becomes noticeable on an aggregate level.

Secondly, the number of beds *per capita* seems to contribute to the increasing rate of treatable mortality. The well-known Roemer’s Law states that a “bed built is bed filled,” in other words, the more hospital beds are available, the higher the use of hospital services, resulting in an overutilization of hospital services (58, 59). Although there is no direct link between Roemer’s Law and hospital mortality, there are some features which may influence hospital mortality, like overcrowded hospital wards and consequent sub-optimal care (60). Our results therefore may confirm that there is sub-optimal care based on the hospital beds indicator and that their availability is not contributing to the reduction of treatable mortality, for men or women.

### 4.3 Strengths and limitations

The major strength of this work is the contribution to the understanding of factors explaining treatable mortality in Europe in women and men and their difference for different levels of mortality rate, which may contribute to reduce the life expectancy gap between women and men. The limitations arising from our analysis derive from the short period considered and the impossibility of establishing causal relationships. It could be argued that contextual and cultural factors might influence treatable mortality, which were not considered due to the difficulty to define those factors into observable and collectable data. Despite the quantile approach used in our analysis, allowing to understand the differences in treatable mortality across countries, it does not directly reflect the effects on life expectancy differences across countries. Finally, the type of health system financing could also be seen as a related factor. However, given that European health systems typically feature universal coverage, either in Beveridge or Bismark-type of health systems, it would be expected to have less relevance in determining treatable mortality, as concluded in another study (24).

## 5 Conclusion

This work provides valuable insights into the factors that influence treatable mortality among both females and males across European countries. Understanding these drivers can support policy making aimed at implementing targeted interventions to reduce treatable mortality rates and improve the overall health in EU countries. There are different kinds of policy recommendations that emerge from this work. First, funding and economic development continue to be strongly associated with health outcomes, specifically in terms of treatable mortality. Secondly, hospital bed management needs to be improved. For instance, implementing scientifically based methods to optimize the allocation of beds in hospitals could improve data on treatable mortality. Finally, addressing the shortage of health professionals requires different responses depending on whether it pertains to nurses or physicians. On the one hand, more physicians ensure a decrease in treatable mortality. On the other hand, improving the understanding of the roles and capabilities of nurses within different hospital settings may also help in reducing mortality rates, rather than exacerbating them.

## Data availability statement

Publicly available datasets were analyzed in this study. This data can be found at: https://ec.europa.eu/eurostat/data/database.

## Author contributions

AT: Writing – original draft, Writing – review & editing.
