# Supplementary material for: Treatable mortality and health care related factors across European countries
Source: Front Public Health. 2024 Feb 16;12:1301825. doi: 10.3389/fpubh.2024.1301825 (PMC10904533; doi:10.3389/fpubh.2024.1301825)
Supplement: Supplementary file 1 [file Table_1.DOCX]

Supplementary Material

Table A1: Selected studies based on panel data models

| **Authors**  **(year)** | **Countries**  **(years)** | **Health outcomes** | **Independent variables** |
| --- | --- | --- | --- |
| Mackenbach et al.  (2019)[10] | 15 European countries  (1990-2015) | Mortality rates and life expectancy by sex | GDPpc; health expenditure; income inequality; education; democracy index; smoking; material deprivation |
| Or  (2000)[13] | 21 OECD countries  (1970-1992) | Potential years of life lost by sex | GDPpc; total health expenditure; public health expenditure; share of white-collar employees; air pollution; alcohol, tobacco, fat, sugar consumption |
| Spijker  (2005)[14] | 43 European countries  (1968-1999) | Mortality rates by sex | GDPpc; government health expenditure; Gini index; education; share of working people in agriculture and industry; divorce rate; alcohol; air pollution; unemployment; urbanization; tobacco, fruit and cereal consumption |
| Arah et al.  (2005)[15] | 18 OECD countries  (1970-1999) | Mortality rate and potential years of life lost | GDPpc; health expenditure; tobacco, alcohol, fat, fruit and veggie, protein consumption; air pollution; physician density; doctors’ visits; share of population over 65 |
| Joumard et al.  (2008)[16] | 22 OECD countries  (1981-2003) | Life expectancy, premature mortality and infant mortality by sex | GDPpc; health spending; tobacco, alcohol, fruit consumption; education; air pollution; physician density |
|  |  |  |  |
| Roffia et al. (2023)[17] | 36 OECD countries (1999-2018) | Life expectancy at birth | GDPpc; health expenditure; out-of-pocket expenditure; physician density; hospital bed density; social spending; participation ratio to labour; prevalence of chronic respiratory diseases; temperature; total size of the population |
| Anwar et al.(2023)[18] | 8 OECD countries  (1996-2020) | Infant mortality and life expectancy | GDP; government health expenditure; number of doctors; population; CO2 emissions |
| Ivankova et al. (2022)[19] | 38 OECD countries (1994-2016) | Treatable mortality due to circulatory system diseases and endocrine, nutritional and metabolic diseases | GDPpc; health care financing |

Table A2: List of countries and treatable mortality rate in 2019

|  | Male | Female |  | Male | Female |  | Male | Female |
| --- | --- | --- | --- | --- | --- | --- | --- | --- |
| Austria | 84.21 | 63.22 | Greece | 114.2 | 74.32 | Netherlands | 59.34 | 63.26 |
| Belgium | 69.09 | 61.35 | Slovakia | 212.62 | 123.08 | Poland | 171.65 | 101.86 |
| Hungary | 227.7 | 130.14 | Iceland | 58.2 | 55.43 | Portugal | 94.16 | 66.3 |
| Croatia | 164.15 | 97.67 | Ireland | 76.98 | 65.17 | Romania | 270.68 | 155.32 |
| Czechia | 154.26 | 90.45 | Italy | 68.13 | 59.8 | Slovenia | 83.24 | 61.52 |
| Denmark | 73.6 | 59.89 | Latvia | 257.97 | 140.46 | Spain | 74.0 | 52.23 |
| Finland | 81.47 | 57.72 | Lithuania | 258.65 | 125.58 | Sweden | 65.35 | 55.14 |
| France | 64.29 | 57.41 | Luxembourg | 71.07 | 55.29 | UK | 95.09 | 80.19 |
| Germany | 94.07 | 70.41 | Malta | 92.38 | 78.3 | Norway | 61.65 | 52.38 |
|  |  |  |  |  |  | Switzerland | 52.02 | 47.54 |

Table A3: Pairwise correlations

|  | Mortality_f | Mortality_m | GDPpc | Health_Exp | Physicians | Nurses | Beds | Hosp_Days_Inpat | ALOSf |
| --- | --- | --- | --- | --- | --- | --- | --- | --- | --- |
| Mortality_m | 0.97 | 1.00 |  |  |  |  |  |  |  |
|  | 0.00 |  |  |  |  |  |  |  |  |
| GDPpc | -0.72 | -0.73 | 1.00 |  |  |  |  |  |  |
|  | 0.00 | 0.00 |  |  |  |  |  |  |  |
| Heath_Exp | -0.60 | -0.64 | 0.50 | 1.00 |  |  |  |  |  |
|  | 0.00 | 0.00 | 0.00 |  |  |  |  |  |  |
| Physicians | -0.27 | -0.19 | 0.09 | 0.15 | 1.00 |  |  |  |  |
|  | 0.00 | 0.00 | 0.14 | 0.02 |  |  |  |  |  |
| Nurses | -0.53 | -0.56 | 0.64 | 0.62 | -0.07 | 1.00 |  |  |  |
|  | 0.00 | 0.00 | 0.00 | 0.00 | 0.31 |  |  |  |  |
| Beds | 0.58 | 0.58 | -0.38 | -0.24 | -0.07 | -0.32 | 1.00 |  |  |
|  | 0.00 | 0.00 | 0.00 | 0.00 | 0.26 | 0.00 |  |  |  |
| Hosp_Days_Inpat | 0.43 | 0.45 | -0.22 | -0.04 | 0.15 | -0.24 | 0.86 | 1 |  |
|  | 0.00 | 0.00 | 0.00 | 0.56 | 0.02 | 0.00 | 0.00 |  |  |
| ALOSf | 0.24 | 0.27 | -0.17 | -0.16 | 0.00 | -0.14 | 0.57 | 0.77 | 1.00 |
|  | 0.00 | 0.00 | 0.01 | 0.01 | 0.95 | 0.04 | 0.00 | 0.00 |  |
| ALOSm | 0.29 | 0.32 | -0.23 | -0.22 | -0.08 | -0.26 | 0.52 | 0.68 | 0.91 |
|  | 0.00 | 0.00 | 0.00 | 0.00 | 0.19 | 0.00 | 0.00 | 0.00 | 0.00 |

Table A4: Values for Shapiro-Wilk test

|  | Shapiro-Wilk W test for normal data | | | |  |
| --- | --- | --- | --- | --- | --- |
| Variable Obs | | W | V | z | Prob>z |
| Mortality_f | 276 | 0.85 | 30.15 | 7.96 | 0.00 |
| Mortality_m | 276 | 0.82 | 36.03 | 8.38 | 0.00 |
| GDPpc | 279 | 0.98 | 3.88 | 3.17 | 0.00 |
| Health_Exp | 261 | 0.96 | 6.63 | 4.41 | 0.00 |
| Physicians | 266 | 0.95 | 8.84 | 5.09 | 0.00 |
| Nurses | 242 | 0.88 | 21.08 | 7.08 | 0.00 |
| Beds | 270 | 0.95 | 9.19 | 5.18 | 0.00 |
| Hosp_Days_Inpat | 248 | 0.98 | 4.27 | 3.38 | 0.00 |
| ALOSf | 248 | 0.98 | 2.88 | 2.46 | 0.01 |
| ALOSm | 248 | 0.98 | 4.43 | 3.46 | 0.00 |

Table A5: Variance Inflation Factors

|  | MALES |  |  |  | FEMALES |  |
| --- | --- | --- | --- | --- | --- | --- |
| Variable | VIF | 1/VIF |  | Variable | VIF | 1/VIF |
|  |  |  |  |  |  |  |
| Hosp_Days_Inpat | 5.95 | 0.1682 |  | Hosp_Days_Inpat | 7.49 | 0.133468 |
| Beds | 4.68 | 0.213583 |  | Beds | 4.92 | 0.203212 |
| ALOSm | 2.05 | 0.4877 |  | ALOSf | 2.66 | 0.375734 |
| Nurses | 1.96 | 0.508942 |  | Nurses | 1.98 | 0.504168 |
| Health_Exp | 1.87 | 0.535842 |  | Health_Exp | 1.84 | 0.543999 |
| GDPpc | 1.78 | 0.563094 |  | GDPpc | 1.78 | 0.561928 |
| Physicians | 1.38 | 0.722625 |  | Physicians | 1.35 | 0.741939 |
|  |  |  |  |  |  |  |
| Mean VIF | 2.81 |  |  | Mean VIF | 3.15 |  |

Table A6: Hausman Test

|  | Females | | Males | |
| --- | --- | --- | --- | --- |
| Test distribution | Statistic | p-value | Statistic | p-value |
| Chi-squared | 16,52 | 0.0112 | -132.99 | chi2<0 |

Note: For Males data fails to meet the asymptotic assumptions of the Hausman test. For Females, H_0_ is rejected, and individual-level effects are adequately modelled by fixed effects.

Table A7: Summary of panel data variables

| Variable | Mean | Std. Dev. | Min | Max | Observations |  |
| --- | --- | --- | --- | --- | --- | --- |
|  |  |  |  |  |  |  |
| Mortality_f | overall | 88.15301 | 32.8812 | 47.54 | 177.74 | N = 276 |
|  | between |  | 32.71971 | 52.05444 | 164.7344 | n = 31 |
|  | within |  | 5.983046 | 72.83079 | 107.5252 | T = 8.90323 |
|  |  |  |  |  |  |  |
| Mortality_m | overall | 132.9157 | 73.96544 | 52.02 | 333.31 | N = 276 |
|  | between |  | 74.27595 | 58.75222 | 293.8533 | n = 31 |
|  | within |  | 9.397051 | 97.43343 | 172.7734 | T = 8.90323 |
|  |  |  |  |  |  |  |
| GDPpc | overall | 10.2148 | 0.377139 | 9.367344 | 11.27594 | N = 279 |
|  | between |  | 0.37071 | 9.510869 | 11.23443 | n = 31 |
|  | within |  | 0.09361 | 9.938674 | 10.50676 | T = 9 |
|  |  |  |  |  |  |  |
| Health_Exp | overall | 6.312031 | 1.822438 | 2.76 | 9.83 | N = 261 |
|  | between |  | 1.792994 | 3.054444 | 9.374444 | n = 31 |
|  | within |  | 0.317111 | 5.147586 | 7.807586 | Tbar = 8.41935 |
|  |  |  |  |  |  |  |
| Physicians | overall | 369.2652 | 77.29162 | 221.27 | 616.12 | N = 266 |
|  | between |  | 75.67351 | 230.2229 | 595.4589 | n = 31 |
|  | within |  | 19.32908 | 305.0364 | 439.4097 | Tbar = 8.58065 |
|  |  |  |  |  |  |  |
| Nurses | overall | 1499.472 | 918.9388 | 400.23 | 3560.69 | N = 242 |
|  | between |  | 918.2368 | 414.8589 | 3454.491 | n = 30 |
|  | within |  | 69.48345 | 1224.216 | 1791.056 | Tbar = 8.06667 |
|  |  |  |  |  |  |  |
| Beds | overall | 496.129 | 166.1122 | 207.03 | 837.84 | N = 270 |
|  | between |  | 167.6539 | 240.9089 | 814.6311 | n = 31 |
|  | within |  | 23.91217 | 395.4645 | 613.3845 | Tbar = 8.70968 |
|  |  |  |  |  |  |  |
| Hosp_Days_Inpat | overall | 124371.7 | 48117.45 | 38829.65 | 242669.8 | N = 248 |
|  | between |  | 46486.65 | 45887.6 | 226728.5 | n = 31 |
|  | within |  | 10757.35 | 88739.44 | 177151 | Tbar = 8 |
|  |  |  |  |  |  |  |
| ALOSf | overall | 7.230645 | 1.520637 | 4.2 | 11.9 | N = 248 |
|  | between |  | 1.45574 | 4.522222 | 9.933333 | n = 29 |
|  | within |  | 0.48551 | 5.218145 | 9.418145 | Tbar = 8.55172 |
|  |  |  |  |  |  |  |
| ALOSm | overall | 7.871371 | 1.330933 | 4.8 | 11.3 | N = 248 |
|  | between |  | 1.255533 | 4.988889 | 10.23333 | n = 29 |
|  | within |  | 0.463322 | 6.302621 | 9.749149 | Tbar = 8.55172 |

Table A8: Quantiles and countries for treatable mortality rate in 2019

| Quantile |  | Quantile value | Countries | (ascending order) | |  |  |  |  |  |  |
| --- | --- | --- | --- | --- | --- | --- | --- | --- | --- | --- | --- |
| <Q20 | Female |  | Switzerland | Spain | Norway | Sweden | Luxembourg | Iceland | Finland |  |  |
|  | Male |  | Switzerland | Iceland | Netherlands | Norway | France | Sweden | Italy |  |  |
| Q20 | Female | 57.7 | Italy | Denmark | Belgium | Slovenia | Austria | Netherlands | Cyprus | Ireland | France |
|  | Male | 68.1 | Belgium | Luxembourg | Denmark | Spain | Ireland | Finland | Slovenia | Austria | Cyprus |
| Q50 | Female | 65.6 | Portugal | Germany | Greece | Malta | UK | Estonia |  |  |  |
|  | Male | 90.8 | Malta | Germany | Portugal | UK | Greece | Czechia |  |  |  |
| >Q70 | Female | 88.5 | Czechia | Croatia | Poland | Slovakia | Lithuania | Hungary | Latvia | Bulgaria | Romania |
|  | Male | 154.3 | Croatia | Poland | Estonia | Slovakia | Hungary | Bulgaria | Latvia | Lithuania | Romania |

Graph A1: Quantile plot for Males Treatable mortality

Graph A2: Quantile Plot for Females Treatable Mortality
